# Educational cartoon as an edutainment strategy to combat pediatric obesity: an innovative proposal from the PODiaCar Project

**DOI:** 10.1186/s13052-025-02082-9

**Published:** 2025-07-17

**Authors:** Gianvincenzo Zuccotti, Valeria Calcaterra, PODiaCar Team, Elvira Verduci, Savina Mannarino, Virginia Rossi, Giulia Fiore, Martina Tosi, Lucia Labati, Matteo Vandoni, Luca Marin, Vittoria Carnevale Pellino, Stefano Conca Bonizzoni, Cristina Campoy, Mireia Escudero Marin, Rocio Bonillo Leon, Lucía Pérez Rodero, Camilo Corbellini, Raphael de Martins Abreu, Khatija Bhadur, Valter Pagani, Umberto Ciriello, Francesco Conti

**Affiliations:** 1 Pediatric Department, Buzzi Children’s Hospital, Milano, Italy; 2 Department of Biomedical and Clinical Sciences, University of Milan, Via GB Grassi, n.74, Milano, 20,157 Italy; 3 Department of Internal Medicine and Therapeutics, University of Pavia, Pavia, Italy

**Keywords:** Educational cartoon, Edutainment strategy, Pediatric obesity, Prevention, PODiaCar

## Abstract

Pediatric obesity is an escalating global health concern with profound physical and psychological impacts, both in the short and long term. The EU-funded initiative, "Fight against Pediatric Obesity: from a Predictive Tool for Type 2 Diabetes and Cardiovascular Disease Risk to Healthy Educational Programs" (PODiaCar), seeks to address childhood obesity through a comprehensive, multidimensional strategy. A key component is a cartoon titled *Grandma Wilma’s Tales*, created to promote healthy lifestyles among young children and their families. Targeted at preschoolers and early primary school students, the cartoon uses an “edutainment” strategy, blending education with entertainment, to keep children engaged while they learn. The story follows a young boy named Vitto and his dog Buzz as they discover the dangers of poor diet and inactivity and the benefits of a healthy, balanced lifestyle. By using simple language, engaging visuals, and relatable characters, the cartoon informs and motivates children to make healthier choices in everyday life. Its design focuses on accessibility and appeal, helping children absorb important health messages in an enjoyable way. This initiative highlights the power of visual communication and storytelling in health education. Teaching children about balanced diets and active living from an early age can build a strong foundation for lifelong wellness. Supporting and sharing projects like PODiaCar at national and international levels is essential for developing future public health strategies and encouraging innovative, child-focused educational approaches.

Pediatric obesity is one of the most pressing public health challenges of our time [1]. According to the World Health Organization (WHO), its prevalence has more than doubled in children aged 2–4 and increased nearly eightfold in those aged 5–19. Currently, about 18.5% of individuals aged 2–19, around 13.7 million children and adolescents, are affected worldwide [2]. As reported by Muyulema et al. [3], without the implementation of effective public health strategies, the WHO’s global nutrition targets for 2025 and the Sustainable Development Goals for 2030 are unlikely to be achieved.

Obesity is recognized as a complex chronic disease, with immediate impacts on the physical and mental health of children and adolescents. It contributes to the early development of serious conditions such as fatty liver disease, cardiovascular disorders and diabetes. Globally, it is one of the leading modifiable risk factors for mortality and disability [4].

Once obesity develops, returning to a normal weight during childhood or adolescence becomes difficult. In fact, in most cases, most rapid weight gain had occurred between 2 and 6 years of age, obesity persists beyond adolescence and leads to further health risks in adulthood [6]. The global rise in childhood obesity results from a complex interplay of genetic predispositions, epigenetic mechanisms, and environmental factors [7]. However, unhealthy eating habits, sedentary lifestyles, and a general lack of awareness about healthy living remain key contributors to this silent epidemic.

Providing high-quality education in health promotion at all levels of schooling represents an effective global investment [8]. In particular, education programs on health promotion and disease prevention starting in early childhood should foster long-term critical thinking processes that encourage positive health choices, behavioral change, and healthy lifestyles [9].

Healthy behaviors established early in life are more likely to persist into adulthood and such behaviors play a key role in preventing or delaying chronic and life-threatening diseases [10]. Encouraging healthy behaviors related to nutritious eating and the importance of physical activity in children can support their development into strong, resilient adults [11].

The role of schools in promoting health has gained support from the WHO’s Ottawa Charter for Health Promotion [12], the anticipated long-term benefits for children and their communities [13], and more recently, research highlighting the positive connection between health and education [14]. The school plays a fundamental role in health education and can serve as a powerful starting point for involving families in disease prevention. Schools are in a unique position to deliver consistent health messages, particularly in communities where family support might be constrained by socioeconomic challenges. Introducing health education in the school setting can help reduce inequalities by ensuring that all children, regardless of family background, have access to dependable and uniform health information.

With regard to lifestyle education, it is important to ensure coherence between the messages children receive at home and at school, as both settings significantly influence their eating behaviors and levels of physical activity [15]. This is especially true for younger children, who depend on adults at home and school for food selection and preparation.

To effectively educate children about healthy lifestyles, it is essential to use tools that can capture and sustain their interest. Innovative digital health education audio-visual models can be employed to teach primary school-aged children about public health, thereby empowering them to adopt healthy behaviors.

As part of the EU-funded initiative "Fight against Pediatric Obesity: from a Predictive Tool for Type 2 Diabetes and Cardiovascular Disease Risk to Healthy Educational Programs" (PODiaCar, EU4H-2022-PJ-3 — EU4H-PJG, Project number 101128946; https://podiacar.eu), we developed an educational cartoon entitled "Grandma Wilma's Tales" as an engaging tool to raise awareness among children about the importance of health, physical activity, and balanced nutrition.

PODiaCar is a project coordinated by Buzzi Children’s Hospital in Milan, Italy, in collaboration with the University of Pavia (Pavia, Italy), the University of Granada (Granada, Spain), LUNEX University of Applied Sciences (Differdange, Luxembourg), and ASOMI College of Science (Malta). The project aims to address childhood obesity and its related complications, such as type 2 diabetes and cardiovascular diseases It aligns with the European Commission’s ‘Healthier Together’ initiative, which targets major non-communicable diseases (NCDs), and fits within the strategic framework of the European Health and Digital Executive Agency (HaDEA). PODiaCar, through multidisciplinary collaboration and digital innovation (educational cartoon, artificial intelligence, digital twins), promotes a proactive, data-driven approach to child health and disease prevention.

One of the main goals of the project is to create a cartoon to launch a widespread awareness campaign promoting healthy lifestyles in schools and families.

The cartoon "Grandma Wilma's Tales" is aimed at preschool children and those in the early years of primary school. It proposes an "edutainment" strategy, combining education with entertainment as an approach to ensure that children remain engaged.

Children are naturally attracted to bright colors, funny characters, and moving animations. This makes cartoons one of the most powerful tools for capturing and maintaining their interest. Children learn by observing the behavior of characters, without feeling like they are being “taught”. Identifying with a positive character who makes healthy choices can inspire them to do the same.

The cartoon has been produced by an animation studio specialized in the creation and production of computer-animated video (Officine Creative, University of Pavia) under the supervision of the project’s scientific coordinator. Essential messages about nutrition and lifestyle were derived from an extensive literature review and refined by a committee composed of pediatricians, experts in pediatric nutrition and adapted physical activity and communication specialists, to ensure they were engaging, age-appropriate, and free from stigmatization.

"Grandma Wilma's Tales" is an animated story centered around Grandma Wilma and her grandson Charlie, who is initially reluctant to adopt healthier eating habits. The episode introduces Vitto and his dog Buzz, and through their story, it explores the negative effects of an unhealthy diet and lack of physical activity, while promoting the benefits of a balanced lifestyle.

To summarize the episode, Charlie loves fried and sugary foods and refuses to eat vegetables. Determined to help him, Grandma Wilma offers a deal, if Charlie agrees to try her vegetable soup, she will tell him a fantastic story. The animated series, featuring Vitto and his blue-haired puppy Buzz, follows their adventures around food, exercise, and health. Grandma Wilma narrates the episodes, guiding viewers through important lessons on nutrition and wellness. In the story, Vitto is a boy who receives Buzz as a birthday gift. The two quickly become inseparable, but over time, Vitto grows lazy and starts gaining weight from unhealthy eating. Buzz notices his friend's low energy and sadness, and tries to help by hiding junk food and encouraging Vitto to be more active. Despite his efforts, Buzz also begins to feel unwell from eating the junk food he had taken from Vitto. With the help of a pediatrician, Vitto realizes that Buzz was only trying to help him live a healthier life. Together, they commit to better habits, eating nutritious food and staying active. Inspired by Vitto’s journey, Charlie decides to try Grandma Wilma’s vegetable soup and discovers it’s not as bad as he expected. Together, he and his grandmother prepare a delicious fruit salad using Grandpa’s fresh fruit, ready to start a healthier chapter in his life.

Through simple language, engaging visuals, and relatable characters, the cartoon aims not only to inform but also to motivate children to make healthy choices in their daily lives. It is a concrete example of how visual communication and play can become powerful allies in the fight against childhood obesity.

The storyline and characters were carefully designed, using clear language and visually appealing graphics to effectively capture children's attention and facilitate learning. By creating an engaging and relatable learning environment, the series motivates children to embrace healthier daily habits.

The entire cartoon can be viewed at the link https://drive.google.com/file/d/1xK25uA8KU53AQ8enDTtD_Ey0c2jc_0i6/view.

The inclusion of new educational resources supports teachers in effectively integrating health programs into schools, while at the same time creating synergy with families. These tools not only enhance knowledge acquisition, but also foster critical thinking and the development of lifelong skills essential for making informed, healthy choices.

Health education from an early age could be particularly impactful when delivered through serialized cartoons, leveraging the familiarity of recurring characters and the strength of evidence-based scientific messages [16]. The use of high-quality, artistically refined animation techniques enables a seamless blend between visual arts and medical language, enhancing both engagement and comprehension.

Educating children about healthy lifestyles from the earliest stages of life means laying a strong foundation for long-term health and well-being. Supporting and promoting educational projects focused on children's well-being, such as PODiaCar, at both national and international levels is essential for sharing innovative ideas that can guide future public health strategies and intervention planning.

## Data Availability

Not applicable.

## Abbreviations

WHO: World Health Organization
NCDs: Non-communicable diseases
HaDEA: European Health and Digital Executive Agency
PODiaCar: Fight against Pediatric Obesity: from a Predictive Tool for Type 2 Diabetes and Cardiovascular Disease Risk to Healthy Educational Programs
